# Evaluation of colonoscopy data for colorectal polyps and associated histopathological findings^☆^

**DOI:** 10.1016/j.amsu.2020.07.010

**Published:** 2020-07-11

**Authors:** Mohammad Kazem shahmoradi, Maryam Soleimaninejad, Masoud Sharifian

**Affiliations:** Department of General Surgery, Faculty of Medicine, Lorestan University of Medical Sciences, Khorramabad, Iran

**Keywords:** Adenomas, Colonoscopy, Polyps, Benign lesions

## Abstract

**Background:**

Adenomas of colon and rectal are frequent colonoscopically found benign lesions. The aim of this study is to evaluate the incidence of polyps among patients referred for colonoscopy and associated histopathological findings.

**Methods:**

In this retrospective study, patients referred for colonoscopy at Shahid Madani Hospital from were enrolled. The records of the patients were evaluated for demographic data, polyp status along with size and location and type and histopathological findings of the polyps. The data obtained were statistically analyzed using SPSSv22.

**Results:**

Among 1600 patients who underwent colonoscopy, 260 were positive with polyps. The main symptom among these patients was lower gastrointestinal bleeding 44.2%. The average size of the polyps was 5.68 ± 2.66 and the incidence was significantly greatest among the age group of 51–65 years, p < 0.01. The commonest sites polyps were sigmoid and descending colon, 28.6% and 23.2%, respectively. Adenomatous polyps were the most frequent type, 58.3%. The morphology and pathology of the polyps were significantly associated with each other, p < 0.01.

**Conclusion:**

Our study evaluates the retrospective data for polyp findings among colonoscopy patients. Early diagnosis of polyps can provide better therapeutic outcomes.

## Highlights

• Adenomas of colon and rectal are frequent colonoscopically found benign lesions.
• Surveillance of colonoscopy could have been compromised since several physicians were included in the study.

## 1 Introduction

Colorectal cancer is one of the most common malignancies, the third most common, and the fourth leading cause of death from cancer worldwide [1]. It accounts for 10% of all cancers in men and 4.9% in women [2]. Over the past 15 years, its prevalence has declined due to screening programs, and mortality rates have dropped up to 10% as a result of early diagnosis and advanced treatment programs [3]. Polyps are benign fleshy or wart-like appendages that grow on the surface of the mucosa [4]. Findings have revealed that colon cancerous tumors can originate from polyps or benign appendages in the mucosal area and take several years to develop from benign to malignant cancer [5]. This process provides an opportunity to prevent colon cancer by early detection of polyps and subsequent treatment [6].

Most colorectal cancers originate from adenomatous polyps, regardless of the cause [7]. Adenomatous polyps may be found in the colon of 30% of middle-aged and 50% of older people, but less than 1% of them become cancerous [8]. Various molecular changes in polyps can lead to malignancy [9]. These changes are point mutations in protooncogene k-ras and DNA hypomethylation - DNA loss on the side of the tumor inhibitor gene (APC: Adenonatous polyposis coil gene in the long arm of chromosome 5), allelic loss on the tumor-gene inhibitor on the chromosome 18q and allelic loss in chromosome p17, which is associated with p53 mutation [10]. Cancers are more common in sessile polyps. Villous adenomatous sessile polyps are 3 times more likely to be sessile than tubular adenoma [11].

The prevalence of colorectal adenomatous polyps varies greatly geographically [12]. Among asymptomatic individuals, the average risk of adenoma prevalence is about 10% in sigmoidoscopic studies and is more than 25% from colonoscopic findings [13].

This aim of this study was to evaluate the frequency of polyps among patients referred to our center for colonoscopy and associated histopathological findings.

## 2 Methods

In this retrospective study, patients referred for colonoscopy at Shahid Madani Hospital from 2015 to 2019 were enrolled. All the patients above the age of 18 who were referred for colonoscopy at outpatient service were included in the study. Patients with incomplete records and those lacking histopathological findings, those with the persona of colorectal cancer, inflammatory bowel disease and colorectal polyps were excluded from the study.

The following data were extracted from patients file: demographic information (age, gender), cause of colonoscopy, biopsy status, number, type and location of polyps, histopathological findings, common complaint (cause of referral). The information obtained were recorded in a checklist for each patient.

The data obtained were computerized and analyzed statistically using SPSSv22. To estimate the frequency of polyps, frequency charts and tables were used and to investigate the relationship between the type of polyps and other parameters, chi-square test and *t*-test methods were used. P value < 0.05 was considered to be statistically significant.

Researchers at all stages of the research adhered to the ethical principles as per Helsinki Declaration and Ethics Council of … (The study was approved by the ethical committee of Lorestan University of Medical Sciences (IR.LUMS.REC.1397.082).).

The names of the patients were not used in the study at any stage and the patients’ information was coded in the form of file numbers.

## 3 Results

This study examined 1600 cases of patients referred to the endoscopy-colonoscopy department for polyp examination. The mean age of patients was 57.92 ± 15.3 years.

As reported in Table 1, the most common cause of the referral of lower gastrointestinal bleeding (GIB), 28.7% followed by chronic constipation 21.2%. Overall, 260 patients were presented with polyps and the average size of the polys was 5.68 ± 2.66. Among patients with polyps, the highest incidence of polyps, 33.5%, were under the age group of 51–65 years, which was statistically significant p < 0.001, Table 2. Furthermore, the incidence of polyps was greater in men than women, 66.2% vs 33.8%. This difference was reported to be statistically significant, p < 0.001.

**Table 1:** Frequency distribution of patients' reasons for endoscopic-colonoscopy.

Reason of referral	Number	Percent	cumulative percentage
Ab pain	164	10.3	10.3
Chronic diarrhea	171	10.8	21.0
chronic constipation	339	21.2	42.2
Anal pain	260	16.3	58.4
Lower GIB	460	28.7	87.2
FH of colon Ca	135	8.4	95.6
IDA	50	3.1	98.8
Hepatic mass	3	0.2	989
OB+	5	0.3	99.3
Screening	5	0.3	99.6
Weight loss	2	0.1	99.6
Gas passage	1	0.1	99.7
Past history of colon polyp	5	0.3	
Total	1600	100.0

**Table 2:** Frequency distribution of demographic characteristics of patients based on the presence or absence of polyps in endoscopy – colonoscopy.

Type of properties		Patients with polyps		Patients without polyps		p-value
		Number	Percent	Number	Percent	
age	20>	15	5.8%	41	3.1	0.001
	21–35	29	11.2	341	25.4	
	36–50	69	26.5	287	21.4	
	51–65	87	33.5	325	24.3	
	65<	60	23.1	346	25.8	
Sex	Male	172	66.2	720	53.7	0.001
	Female	88	33.8	621	46.3	
Total		260	100	1340	100	
Age mean		MEAN ± SD		MEAN ± SD		
		14 ± 59.12		15.1 ± 52.37		

The most common cause of referral among polyp positive patients was lower GIB and chronic constipation. Among these two groups, 44.2% and 23.1% patients were positive with polyps, respectively.

The most common sites of polyps were sigmoid colon 28.6%, descending colon 23.2% and rectum 22.8%. Additionally, 86.5% of the patients had one polyp and 10.8% were presented with two polyps. 51.7% patients had sessile while 48.3% had pedunculate polyps. Adenomatous polyps were pathologically the most common type, reported among 58.3% patients, followed by hyperplastic polyps, 25.1%. The pathology of the polyp was not significantly associated with the site of polyp, p = 0.4.

In adenomatous polyps, the most common morphology was sessile 58.3% and in pedunculate polyps, hyperplastic type 60% was the most common one. However, pathology of the polyp was significantly correlated with the morphology of the polyp, p = 0.01 (Table 3).

**Table 3:** Distribution of polyp morphology in endoscopy - colonoscopy based on pathology.

	Polyp pathology						Total	p-value
		adenomatous	hyperplastic	pesudopolyp	tubulo-villous	juvenile		
Pedunculate	Number	63	39	6	13	4	125	0.01
	Percent	41.7	60	100	50	36.4	48.3	
sessile	Number	89	26	0	13	7	135	
	Percent	58.3	40	0	50	63.6	51.7	
Total	Number	152	65	6	26	11	260	
	Percent	100	100	100	100	100	100	

## 4 Discussion

The results of our study indicated that the most common causes of patients’ referral with polyps was lower gastrointestinal bleeding. In a study by Jahangiri et al. among 150 patients 14.3% patients with polyps were presented with lower GIB [14]. Similarly, in another study regarding age-related symptoms and manifestations of familial adenomatous polyps, most of these patients were reported to have intestinal symptoms such as colon bleeding (68%) and diarrhea (42%). These findings are consistent with those from our study. However, a report by Adelstein et al. on the symptoms of polyps, colon and rectal cancer showed no weight loss and lower gastrointestinal bleeding among these patients [15]. Therefore, given the conflicting results, lower gastrointestinal bleeding can be considered one of the most important non-diagnostic causes and symptoms of colorectal polyps.

The outcomes of our study showed that, of 1600 patients who underwent colonoscopy, 260 were seen to have polyps and the average size of the polyps was 5.68 ± 2.66. In a study by Delvari et al., 5427 colonoscopies were performed. Polyps were more common in patients after the age of 60 year and 2.9% of men and 1.9% of women were diagnosed with polyps. 12.5% of these patients were presented with gastrointestinal bleeding. In a study by Amini et al., among patients referred to Imam Khomeini Hospital, Tehran for colonoscopy, of 1172 patients 200 (17.1%) had polyps in colon. The results from the study showed that 1 of every 5.8 patient was presented with polyps, which is approximately similar to findings from our study [16].

Our study reported that the incidence of polyps was significantly higher among the age ≥65 years and among male gender, p < 0.05. In a study conducted in Rawalpindi, Pakistan, polyps were more common in men than women (63.2% vs. 53.7%) [17]. Among 436 patients included, 75.9%, men and 24.1% women were with an average age of 55 years. These findings are in parallel with our studies. Similar findings have also been reported by Azarhoush et al. [18].

The most common site of polyp was colon and rectum, in our study. Qumseya et al. performed colonoscopy among 2400 patients (50.5% female and 49.5% male). Of 51.87% patients presented with polyps, 54% (1636) had polyps on the right and 46% (n = 1409) on the left side of the colon [19]. The adenoma polyps in the right of colon was significantly higher than in the left: 69.4% versus 39.3%. In a study by Emadian et al. [20], 54% of the polyps were in the rectosigmoid region, which is consistent with our results.

Azharhoush et al. also reported that 51.7% of the polyps were sessile and were the most common type of adenoma, which is similar to our outcomes and those by Ma and colleague [21]. In a study conducted by Akhlaqi and his colleagues at Ghaem Medical Center in Mashhad, among 145 patients with gastrointestinal polyps, 52.7% had juvenile polyps, 4.5% had adenomatous polyps, 12.7% inflamed polyps, and 10% had hyperplastic type [22]. However, in our study, adenomatous and hyperplastic polyps were the commonest ones. In a study conducted at Cochin Hospital in India, the prevalence of polyps was 11% where hyperplastic polyps were 5.49%, adenomatous 8.41% and inflammatory were 12.6% [23]. These differences may be due to the small sample size compared to our study. Similarly, Valarini and coworkers conducted 2401 colonoscopies where 24.3% were positive for polyps. 60% of these patients were female and the mean age of the patients was 58 ± 12 years [24]. The common sites of polyps were colon (38.5%) and rectum (32.5%) and histologically, tubular adenomas, hyperplastic and serrated polyps had greatest incidence, respectively.

## 5 Conclusion

The results of the study are restricted to single center and retrospectively recorded, therefore, does not apply to general population. Several risk factors such as smoking, diet, medicines and protective factors like NSAIDs (non-steroidal anti-inflammatory drugs) were also not included in the study. Despite the colonoscopists were highly trained and experienced, surveillance of colonoscopy could have been compromised since several physicians were included in the study.

Our study provides the colonoscopy findings associated with polyps. Early diagnosis and therapeutic measurements for colorectal cancer can increase the survival rate and provide better quality of the life.

## Ethical approval

All procedures performed in this study involving human participants were in accordance with the ethical standards of the institutional and/or national research committee and with the 1964 Helsinki Declaration and its later amendments or comparable ethical standards.

## Funding

No funding was secured for this study.

## Author contribution

Dr. Mohammad Kazem shahmoradi: conceptualized and designed the study, drafted the initial manuscript, and reviewed and revised the manuscript.

Dr. Maryam Soleimaninejad: Designed the data collection instruments, collected data, carried out the initial analyses, and reviewed and revised the manuscript.

Dr. Masoud Sharifian: Coordinated and supervised data collection, and critically reviewed the manuscript for important intellectual content.

## Registration of research studies

Name of the registry: Lorestan University of Medical Sciences.

Unique Identifying number or registration ID: (IR.LUMS.REC.1397.082).

Hyperlink to the registration (must be publicly accessible): http://ethics.research.ac.ir/ProposalViewEn.php?id=27102.

## Guarantor

Mohammad Kazem Shahmoradi.

## Provenance and peer review

Not commissioned, externally peer reviewed.

## Human and animal rights

No animals were used in this research. All human research procedures followed were in accordance with the ethical standards of the committee responsible for human experimentation (institutional and national), and with the Helsinki Declaration of 1975, as revised in 2013.

## Consent for publication

Informed consent was obtained from each participant.

## Availability of data and materials

All relevant data and materials are provided with in manuscript.

## Declaration of competing interest

The authors deny any conflict of interest in any terms or by any means during the study.

## References

[1] Mozafar Saadati H., Khodamoradi F., Salehiniya H. (2020). Associated factors of survival rate and screening for colorectal cancer in Iran: a systematic review. J. Gastrointest. Canc..

[2] Akhoond M.R., Kazemnejad A., Hajizadeh E., GAnbary Motlagh A., Zali M.R. (2011). Comparison of influential factors affecting survival of patients with colon and rectum cancer using competing risks model. Koomesh.

[3] Roudbari M., Abbasi A.S.L.M., Barfei F., Gohari M.R., Khodabakhshi R. (2015). Survival analysis of colorectal cancer patients and its prognostic factors using cox regression. Razi J. Med. Sci..

[4] Cancer IAfRo (2008). Colorectal Cancer Incidence and Mortality Worldwide in 2008.

[5] Macrae F., Bendell J., Tanabe K., Savarese D., Grover S. (2017). Clinical Presentation, Diagnosis, and Staging of Colorectal Cancer.

[6] Byrne R.M., Tsikitis V.L. (2018). Colorectal polyposis and inherited colorectal cancer syndromes. Ann. Gastroenterol..

[7] He X., Wu K., Ogino S., Giovannucci E.L., Chan A.T., Song M. (2018). Association between risk factors for colorectal cancer and risk of serrated polyps and conventional adenomas. Gastroenterology.

[8] Wilkins T., McMechan D., Talukder A. (2018). Colorectal cancer screening and prevention. Am. Fam. Physician.

[9] Click B., Pinsky P.F., Hickey T., Doroudi M., Schoen R.E. (2018). Association of colonoscopy adenoma findings with long-term colorectal cancer incidence. Jama.

[10] Jover R., Dekker E., Schoen R., Hassan C., Pellise M., Ladabaum U. (2018). WEO expert working group of surveillance after colonic neoplasm. Colonoscopy quality requisites for selecting surveillance intervals: a world endoscopy organization delphi recommendation. Dig. Endosc..

[11] Kang H., Thoufeeq M.H. (2018). Size of colorectal polyps determines time taken to remove them endoscopically. Endosc. Int. Open.

[12] Gibson D.J., Nolan B., Rea J., Buckley M., Horgan G., Sheahan K. (2018). A prospective study of faecal immunochemical testing following polypectomy in a colorectal cancer screening population. Frontline Gastroenterol..

[13] Arana-Arri E., Imaz-Ayo N., Fernández M.J., Idigoras I., Bilbao I., Bujanda L. (2018). Screening colonoscopy and risk of adverse events among individuals undergoing fecal immunochemical testing in a population-based program: a nested case-control study. Unit. Eur. Gastroenterol. J..

[14] Gul R., Hadayat R., Khan A.N., Khursheed L. (2017). Causes of lower gastrointestinal bleeding on colonoscopy. J. Ayub Med. Coll. Abbottabad.

[15] Adelstein B.-A., Macaskill P., Chan S.F., Katelaris P.H., Irwig L. (2011). Most bowel cancer symptoms do not indicate colorectal cancer and polyps: a systematic review. BMC Gastroenterol..

[16] Amini A.Q., Samo K.A., Memon A.S. (2013). Colorectal cancer in younger population: our experience. J. Pakistan Med. Assoc..

[17] Akhtar M.R., Ishaque M., Saadat U. (2004). Aetiology of nasal polyp–a study of 200 cases at combined military hospital Rawalpindi. Pakistan J. Otolaryngol..

[18] Azarhoush R., Amiriani T., Rahimi-Nejad N. (2014). Anatomical and histopathological distribution of gastrointestinal polyps in Gorgan, Iran (1999-2008). J. Gorgan Univ. Med. Sci..

[19] Qumseya B.J., Coe S., Wallace M.B. (2012). The effect of polyp location and patient gender on the presence of dysplasia in colonic polyps. Clin. Transl. Gastroenterol..

[20] Emadian O., Torabizadeh Z., Taheri H. (2016). Epidemiologic, colonoscopic and histologic characteristics of colorectal polyps. J. Mazandaran Univ. Med. Sci..

[21] Ma M.X., Bourke M.J. (2017). Sessile serrated adenomas: how to detect, characterize and Resect. Gut and Liver.

[22] Sharifi D.D., Akhlaghi M. (2000). Review of 145 Patients with Gastrointestinal Polyps of Ghaem Medical Center.

[23] Jayadevan R., Anithadevi T., Venugopalan S.S.R.P. (2016). Prevalence of colorectal polyps: a retrospective study to determine the cut-off age for screening. J. Gastroenterol. Pancreatol. Liver Disord..

[24] Valarini S.B.M., Bortoli V.T., Wassano N.S., Pukanski M.F., Maggi D.C., Bertollo L.A. (2011). Correlation between location, size and histologic type of colorectal polyps at the presence of dysplasia and adenocarcinoma. J. Coloproctol. (Rio J.).

